# Quantitative Assessment of Intervertebral Disc Degeneration Using the Disc Signal Intensity Index in Patients With Spine-Related Pain

**DOI:** 10.7759/cureus.63553

**Published:** 2024-07-01

**Authors:** Koki Tsuchiya, Ichiro Okano, Reon Kobayashi, Yusuke Dodo, Joji Ogawa, Eiko Hara, Asae Taketomi, Yoshifumi Kudo

**Affiliations:** 1 Department of Orthopedic Surgery, Showa University School of Medicine, Tokyo, JPN; 2 Department of Anesthesiology, Showa University School of Medicine, Tokyo, JPN

**Keywords:** i̇ntervertebral disc degeneration, quality of life, euroqol group 5 dimension 5-level quality of life, spine-related pain, disc signal intensity index

## Abstract

Objective

This is a retrospective observational study that aims to investigate the association between disc signal intensity index (DSI2) scores and patient-reported outcome measures in patients with lumbar spine disorders.

Methods

We introduced DSI2 to quantitatively assess disc degeneration. MRI records of patients with lumbar spine-related pain between 2019 and 2022 were analyzed retrospectively. Patient demographics and outcomes were collected, including the Numerical Rating Scale of Pain and EuroQol Group 5 Dimension 5-Level Quality of Life (EQ-5D-5L) scores. The DSI2 was calculated by dividing the mean signal intensity of the L1-S1 discs by that of the CSF on midsagittal T2-weighted MRI images.

Results

Each DSI2 level corresponded to a Pfirrmann grade score at the respective lumbar level. Multivariable linear regression analysis using the EQ-5D-5L as the objective variable identified BMI (p = 0.007) and average DSI2 (p = 0.018) as independent risk factors for EQ-5D-5L deterioration. However, the mean Pfirrmann grade score was not an independent risk factor (p = 0.58).

Conclusion

Our study using DSI2 showed the relationship between disc degeneration and EQ-5D-5L deterioration. Distinct from the Pfirrmann grading system, the DSI2 method is a promising alternative for future disc research that excellently detects the subtle progression of degeneration.

## Introduction

MRI is the standard imaging modality for diagnosing lumbar degenerative disc disorders. MRI-based disc evaluation for assessing degenerative disc conditions started approximately concurrently with the clinical application of MRI [1]. The Pfirrmann grading scale is an MRI-based assessment, first reported in 2001, that utilizes the signal characteristics of discs in T2-weighted MRI images, which reflect the water content, and classifies discs on a five-point scale (from one [A1] to five) [2].

The Pfirrman grading scale is one of the most frequently used disc degeneration classifications, exhibiting good reproducibility [3,4]; however, one criticism argues that the grading system has only five grades and may thus not be granular enough to properly assess disc conditions. Griffith et al. reported an eight-level modified grading system for assessing disc degeneration [5], in which the additional modified grades are subdivisions of Grades 4 and 5 in the Pfirrman grading scale. Consequently, early-stage evaluation using the modified grading system remained unchanged. Moreover, whether these classifications reflect patient-reported outcomes such as quality of life (QOL) scores remains unclear. Disc degeneration is also quantitatively evaluated. An example is the apparent diffusion coefficient (ADC), which quantitatively measures the movement of water molecules in tissues. However, ADC requires special MRI sequencing and thus cannot be measured retrospectively [6].

An MRI-based quantitative bone strength assessment, the vertebral bone quality (VBQ), was recently introduced [7]. The VBQ score reportedly predicts fragility fractures independent of bone mineral density [8]. Of note, VBQ assessment can be performed retrospectively using previously captured images and requires no special software or imaging sequences. We applied the concept of VBQ to assess intervertebral disc degeneration and developed a disc signal intensity index (DSI2) by measuring the signal intensity at three locations in the intervertebral disc on MRI T2-weighted images and dividing the average value by the CSF signal intensity. This study aimed to investigate the association between DSI2 scores and patient-reported outcome measures in patients with lumbar spine disorders.

This article was not previously presented as a meeting abstract.

## Materials and methods

Data collection

This study was conducted at Showa University School of Medicine, Tokyo, Japan. An institutional ethics board approval was obtained for this study (approval number 2023-169-A). The requirement for informed consent was waived because of the retrospective nature of the study. The electronic records of patients who visited our pain clinic between 2014 and 2016 were retrospectively reviewed. Patients who underwent lumbar MRIs but did not undergo lumbar spinal surgery were included in the study. The exclusion criteria were as follows: suspected spinal infection (pyogenic vertebral discitis, osteomyelitis, or both), spinal oncological conditions, or prolonged post-procedure pain syndrome after a spinal procedure such as epidural injection. Patient demographics, including age, weight, height, BMI, Numerical Rating Scale of Pain, and EuroQol Group 5 Dimension 5-Level Quality of Life (EQ-5D-5L) scores, were obtained through the routinely administered baseline questionnaire.

DSI2 measurement

**Figure 1:**
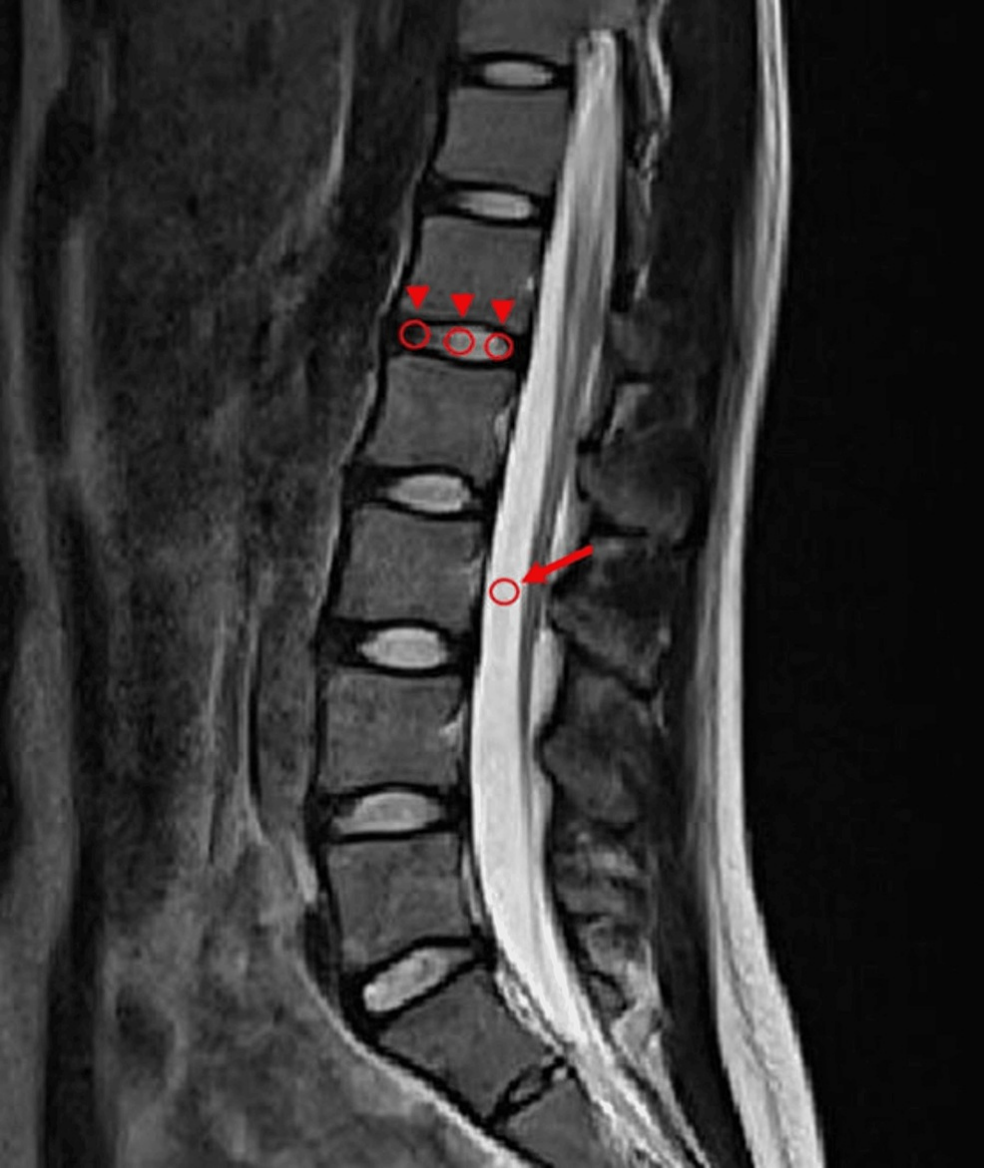
Representative ROI measurements of the disc and CSF signal intensities on T2-weighted midsagittal MRI images. Disc signal intensities were measured in three regions at the L1/2 disc (arrowhead) along with the ROI of CSF signal intensity (arrow). ROI, region of interest; CSF, cerebrospinal fluid; MRI, magnetic resonance imaging

DSI2 measurements were performed on midsagittal T2-weighted MRI images by determining the intensity within the regions of interest (ROIs) (Figure 1). One ROI was set in the CSF, whereas three ROIs were set in the anterior, middle, and posterior third portions of each disc. First, the middle ROI was set at the center of the disc, and anterior and posterior ROIs were positioned at each quantile point. The diameters of the ROIs matched the heights of the discs at each position. The endplates were completely excluded from ROIs. For CSF ROI placement, an area posterior to the L3 vertebra without the descending nerve roots was selected. In cases of spinal stenosis at the L3 level, due to the lack of available free space for performing the CSF measurement, the next possible level (L2 or L4) was selected. The endplates were completely excluded from ROIs. The DSI2 score was calculated by dividing the mean of the three measurements per disc by that of the CSF. This process was completed for the L1/L2-L5/S1 discs. MRI images were independently assessed by two board-certified orthopedic surgeons blinded to the background information of participants.

Statistical analysis

Categorical variables were described as counts and percentages. The mean (SD) or median (range) was used for continuous variables, depending on the normality of the distributions. Chi-square or Fisher’s exact tests were used to compare categorical variables. The Shapiro-Wilk test was performed to assess the normality of continuous variables. The Mann-Whitney U or Wilcoxon signed-rank test was used to compare continuous variables because specific continuous variables had nonnormal distributions. The Bonferroni adjustment for multiple comparisons was performed for the comparisons of DSI2 between each Pfirrmann grade. The interclass correlation coefficients (ICC) were calculated for interobserver agreement. An ICC value of <0.50 indicated poor agreement, 0.50-0.75 indicated moderate agreement, 0.75-0.90 indicated good agreement, and >0.90 indicated excellent agreement. Statistical significance was set at two-tailed p-values <0.05. Statistical analyses were performed using the R software (version 4.1.2; R Foundation for Statistical Computing, Vienna, Austria).

## Results

In total, 33 patients and 151 discs were included in the final analysis. The mean (SD) age was 41.5 (21.69) years, and 48.5% of patients were women. Of the 33 patients, 69.7% presented with leg pain, and 66.7% presented with back pain.

Table 1 presents the clinical characteristics of the patients. The mean (SD) BMI was 24.5 (4.6). Three patients (9.1%) were diagnosed with degenerative disc disease, eight (24.2%) with lumbar spinal canal stenosis, and 22 (66.7%) with lumbar disc herniation.

**Table 1:** Patient demographics EQ-5D-5 L, EuroQol Group 5 Dimension 5-Level Quality of Life; DDD, degenerative disc disease; LCS, lumbar canal stenosis; LDH, lumbar disc herniation; NRS, Numerical Rating Scale

Factor	Subgroup	Description	Number (n = 33)
Age, years		Mean (SD)	41.5 (21.7)
Sex	Female	n (%)	16 (48.5)
BMI, kg/m^2^		Mean (SD)	24.54 (4.57)
Primary diagnosis	DDD	n (%)	3 (9.1)
	LCS	n (%)	8 (24.2)
	LDH	n (%)	22 (66.7)
Nerve compression confirmed with an MRI		n (%)	25 (75.8)
Back pain		n (%)	22 (66.7)
Leg pain		n (%)	23 (69.7)
Psychiatric history	None	n (%)	28 (84.8)
	Depression	n (%)	1 (3.0)
	Panic disorder	n (%)	2 (6.1)
	Schizoaffective disorder	n (%)	1 (3.0)
	Schizophrenia	n (%)	1 (3.0)
Current treatment	Opioid	n (%)	0 (0.0)
	Tramadol	n (%)	18 (54.5)
	Epidural steroid injection	n (%)	21 (63.6)
NRS		Mean (SD)	6.64 (2.25)
EQ-5D-5L		Mean (SD)	0.53 (0.23)

The mean (SD) EQ-5D-5L score at baseline was 0.53 (0.23). Table 2 shows the Pfirrmann grade distribution on MRI results. The mean (SD) DSI2 was 0.56 (NA) in Pfirrmann Grade 1 discs, 0.44 (0.14) in Grade 2 discs, 0.33 (0.13) in Grade 3 discs, 0.16 (0.07) in Grade 4 discs, and 0.15 (0.08) in Grade 5 discs.

**Table 2:** Distribution of DSI2 scores among the different Pfirrmann grades DSI2, disc signal intensity index; NA, not applicable

Disc level	Description	Pfirrmann grade				
		1	2	3	4	5
L1/2	n (%)	0 (0.0)	12 (36.4)	17 (51.5)	4 (12.1)	0 (0.0)
L2/3	n (%)	1 (3.0)	9 (27.3)	13 (39.4)	9 (27.3)	1 (3.0)
L3/4	n (%)	0 (0.0)	8 (24.2)	11 (33.3)	13 (39.4)	1 (3.0)
L4/5	n (%)	0 (0.0)	5 (15.2)	8 (24.2)	12 (36.4)	8 (24.2)
L5/S1	n (%)	0 (0.0)	6 (18.2)	5 (15.2)	12 (36.4)	10 (30.3)
DSI2	Mean (SD)	0.56 (NA)	0.44 (0.14)	0.33 (0.13)	0.16 (0.07)	0.15 (0.08)

**Figure 2:**
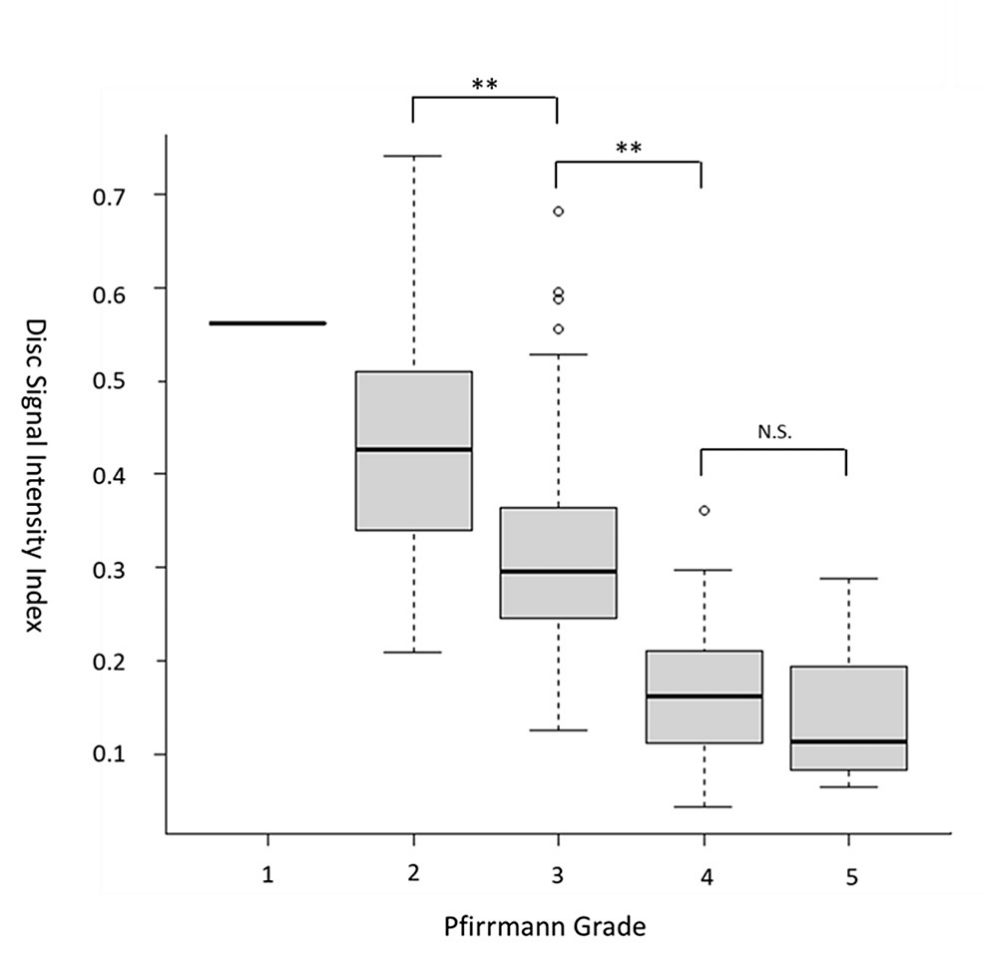
Relationship between DSI2 scores and Pfirrmann grades The box represents the interquartile range, with the line indicating the median and whiskers showing the 10th and 90th percentiles. ** p < 0.01 (with the Bonferroni adjustment for multiple comparisons) DSI2, disc signal intensity index

We observed significant differences in the DSI2 scores between Grade 2 or 3 and Grade 4 or 5 discs (p < 0.01), whereas no significant difference was found between Grade 4 and 5 discs (Figure 2).

In total, we used 151 discs to assess the interobserver agreement using the class correlation coefficient (ICC). We found excellent interobserver reliability for DSI2 scores with an ICC of 0.95 (95% CI: 0.93-0.96). Multivariate logistic regression analysis using the EQ-5D-5L score as the objective variable revealed that the BMI (p = 0.007) and average DSI2 of all discs (p = 0.018) were independent risk factors for EQ-5D-5L deterioration. However, the differences in the mean Pfirrmann grade were not significant (Table 3).

**Table 3:** Results of multivariate analyses for EQ-5D-5L deterioration * indicates statistical significance (p < 0.05). DSI2, disc signal intensity index; EQ-5D-5L, EuroQol Group 5 Dimension 5-Level Quality of Life

Model	Factors	Regression coefficient	95% CI	p-value
Model 1 (average Pfirrmann)	Age	0	-0.01	0.96
	BMI	-0.03	-0.04	<0.001*
	Average Pfirrmann grade	-0.05	-0.1	0.58
	Adjusted R^2^ = 0.355			
Model 2 (DSI2)	Age	0	0.00-0.00	0.96
	BMI	-0.03	-0.04	0.007*
	Average DSI2	0.83	0.16-1.51	0.018*
	Adjusted R^2^ = 0.432			

## Discussion

This study introduced DSI2 as a quantitative measure of disc degeneration based on the signal intensity of the disc on midsagittal T2-weighted MRI images. In our study, DSI2 values demonstrated a linear correlation to the Pfirrmann grading scale, making them particularly useful for distinguishing early stages of disc degeneration, such as Grade 2 or 3. Furthermore, the DSI2 score was significantly associated with patient-reported QOL scores independent of BMI and age, whereas the Pfirrmann grade score did not show a significant association.

The intervertebral disc has three distinct components: the central gelatinous nucleus pulposus, the outer annulus fibrosus, and the cartilaginous endplate that anchors onto the vertebral body [4]. Disc degeneration involves age-related changes and tissue damage caused by multiple stresses [9,10], leading to the loss of proteoglycans and collagen type II [11]. The diagnosis of disc degeneration is usually based on MRI findings, with signal loss in T2-weighted images reflecting reduced proteoglycan and water content in the disc [1,4,5,12]. However, a previous study reported large intersubject variability in the magnitude of signal intensity on T2-weighted images [13]. The VBQ methodology defines the VBQ score as the median signal intensity of L1-4 vertebral bodies divided by the signal intensity of the CSF [7,14], which is used to standardize the vertebral signal intensity values. In our study, aligning with the VBQ assessment methodology, we also utilized CSF as a signal intensity reference, which might have made DSI2 more predictive of EQ-5D-5L scores than the average Pfirrmann grade score that is likely influenced by baseline MRI image conditions.

In previous studies, the ROIs for disc signal intensity measurements were defined as the whole or central part of the nucleus pulposus and did not include the annulus fibrosus [13,15,16]. However, the signal intensity is not constant, even in the nucleus. More specifically, in their study on cervical intervertebral discs, Liu et al. reported that the signal intensity was higher in the central part of the nucleus pulposus than in the anterior and posterior parts [17]. Moreover, clearly differentiating between the nucleus and annulus fibrosus is often difficult, particularly at later stages of disc degeneration. Therefore, we defined the ROIs as the average of three intervertebral disc sites, including the nucleus pulposus and annulus fibrosus. Our study attempted to overcome the limitations of previous studies that focused solely on the nucleus pulposus by adopting a simple trisection method for intervertebral discs. Accordingly, this comprehensive approach provides a detailed understanding of disc degeneration and accurately assesses the dynamic interplay between its structural components.

Previous studies reported that individuals without prior spine surgery or disc degeneration demonstrated significantly higher EQ-5D-5L scores than those with these conditions [18]. In this study, the average DSI2 score was an independent risk factor for EQ-5D-5L deterioration, whereas the average Pfirrmann grade score was not. These findings suggested that the disadvantages associated with the Pfirrmann grade can be mitigated using DSI2. Furthermore, the DSI2 method is a promising alternative for future disc research, excellently detecting subtle degenerative progression and distinguishing itself from the Pfirrmann grading scale.

The present study had certain limitations. First, it was a retrospective study with a small number of patients. One reason was that we excluded all patients who had had previous lumbar spine surgery. Owing to limited data availability, we could not include other potential factors contributing to disc degeneration, such as medical comorbidities and osteoporosis treatment. Second, DSI2 provides useful information for evaluating disc degeneration; however, reduced disc height, usually observed in patients with severe disc degeneration, was not considered in this study, and thus the disc height needs to be separately assessed in future research. Also, we did not involve musculoskeletal radiologists due to availability issues as well as our purpose, which was to introduce a relatively easy method that anyone can objectively measure. Finally, despite the potential advantage of DSI2 in longitudinal assessments, it is essential to note that our current investigation was a cross-sectional assessment. Finally, despite the potential advantage of DSI2 in longitudinal assessments, it is essential to note that our current investigation was a cross-sectional assessment.

## Conclusions

The present study using DSI2 provides valuable insights for identifying the association between disc degeneration and EQ-5D-5L deterioration. The DSI2 method has emerged as a promising alternative for future disc research, excelling at detecting the subtle progression of disc degeneration and distinguishing itself from the Pfirrmann grading system. These findings contribute to an improved and objective understanding of lumbar disc degeneration and may facilitate future research in this field.
